# ReFIT study (reversing frailty in transplantation): protocol for a longitudinal study to assess clinical and biomedical changes in frailty through kidney transplantation

**DOI:** 10.1136/bmjopen-2025-100158

**Published:** 2026-03-02

**Authors:** Trent Payne, Alyra Shaw, Leila Shafiee Hanjani, Ryan Homes, Fiona Giddens, Halley Gora Ravuri, Chloe X Yap, James Walsh, Vinod Kumar, Fleur C Garton, Handoo Rhee, Alan Huang, Ross S Francis, Natasha Reid, Mara McAdams-DeMarco, Emily Gordon, Mark Midwinter, Ruth Hubbard

**Affiliations:** 1 Australian Frailty Network, Centre for Health Services Research, Faculty of Medicine, The University of Queensland, Brisbane, Queensland, Australia; 2 School of Biomedical Science, The University of Queensland, Brisbane, Queensland, Australia; 3 Department of Nephrology, Metro South Hospital and Health Service, Woolloongabba, Queensland, Australia; 4 The University of Queensland Faculty of Natural Resources Agriculture and Veterinary Science, Gatton, Queensland, Australia; 5 Metro South Addiction and Mental Health Services, Metro South Health Service District, Woolloongabba, Queensland, Australia; 6 Mater Research Institute-UQ, South Brisbane, Queensland, Australia; 7 Heart Lung Institute, The Prince Charles Hospital, Chermside, Queensland, Australia; 8 School of Allied Health, Griffith University Griffith Health, Gold Coast, Queensland, Australia; 9 Institute for Molecular Bioscience, The University of Queensland, Brisbane, Queensland, Australia; 10 The University of Queensland, Brisbane, Queensland, Australia; 11 Department of Urology and Kidney Transplantation, Metro South Hospital and Health Service, Woolloongabba, Queensland, Australia; 12 Department of Kidney and Transplant Services, Princess Alexandra Hospital, Woolloongabba, Queensland, Australia; 13 Faculty of Medicine, The University of Queensland, Brisbane, Queensland, Australia; 14 NYU Grossman School of Medicine and Langone Health, NYU Langone Health, New York City, New York, USA; 15 Geriatric Medicine, Metro South Hospital and Health Service, Woolloongabba, Queensland, Australia; 16 General and Geriatric Medicine, Princess Alexandra Hospital, Woolloongabba, Queensland, Australia

**Keywords:** Frailty, Renal transplantation, Aging, GERIATRIC MEDICINE

## Abstract

**Abstract:**

**Introduction:**

Losses of functional reserve across multiple physiological systems have been identified in frail patients, yet the exact aetiology of frailty remains unclear. Although strongly associated with chronological age, frailty often develops at a younger age in patients with organ failure. Frailty is prevalent in patients with kidney failure; however, individuals experience improvements in physical frailty measures following kidney transplantation. This makes younger patients with kidney failure a unique population for studying both the accelerated onset of frailty and its reversal. This research project aims to test the hypothesis that frailty secondary to organ failure and age-related frailty are associated with similar molecular and physiological measures.

**Methods and analysis:**

This longitudinal study will recruit 150 patients in three groups. Group A (kidney transplant recipients aged ≥40 years; n=50) and Group B (patients aged ≥40 years active on the kidney transplant waitlist; n=50) will comprise younger adults with frailty from organ failure. Group C (adults aged ≥65 years (or ≥55 years for Aboriginal and Torres Strait Islander patients); n=50) will comprise older community dwellers. The primary outcome is the Frailty Index (FI). Secondary outcomes include the change in FI over time, and at baseline when considering various clinical metadata, immune parameters, kidney function and nutrition intake which will be measured at baseline and 12-month time points. Longitudinal changes in frailty will be analysed using linear mixed models with multiple testing corrections for false discovery rates.

Endocrine profiles and metabolomics, measures of immune function and microcirculatory dysfunction, will be measured by liquid chromatography-mass spectrometry and/or gas chromatography-mass spectrometry. The gut microbiome will be sequenced via shotgun metagenomics (Illumina NextSeq500, 150 bp paired-end, ^3^Gbp/sample). Circulating cell-free DNA/mitochondrial DNA will be quantified through droplet digital PCR. Microcirculation will be assessed via sublingual dark field videomicroscopy with glycocalyx markers measured by ELISA.

**Ethics and dissemination:**

This study will be conducted with all stipulations of this protocol, and the conditions of the ethics committee approval. Ethical principles have their origin in the Declaration of Helsinki, all Australian and local regulations and in the spirit of the standard of Good Clinical Practice (as defined by the International Conference on Harmonisation). Organs/tissues will be sourced ethically and will not be sourced from executed prisoners or prisoners of conscience or other vulnerable groups.

Ethics approval was received by the Metro South Health Research Ethics Committee (HREC/2023/QMS/95392) and ratified by the University of Queensland.

Results will be disseminated through peer-reviewed publications, academic conferences, participant newsletters and health organisation collaboration.

**STRENGTHS AND LIMITATIONS OF THIS STUDY**

- This study uses comprehensive molecular profiling including metabolomics, microbiomics, endocrine measures and microcirculatory assessment to examine their associations with frailty status, using gold standard experimental techniques (liquid chromatography-mass spectrometry/metagenomic sequencing/digital droplet PCR) to provide high-quality biological data.
- Three-group comparative design (transplant recipients, dialysis patients, older adults) enables differentiation between several aspects of disease-specific and age-specific molecular and physiological characteristics of frailty.
- Longitudinal paired design with baseline measures immediately pre-transplantation maximises ability to detect within-person changes and minimises confounding from temporal variation in frailty status.
- Heterogeneity in frailty pathways among participants and measuring outcomes only at baseline and 12 months may limit the ability to identify universal mechanisms and overlook critical intermediate changes in frailty status.
- Baseline data collection for deceased donor recipients occurs during the preoperative period, which may be stressful and may result in some missing data for certain measures (eg, stool samples); however, pilot experience demonstrates this as feasible for most core measures.

## Introduction

 Frailty represents a significant national and global concern. It is a state of diminished physiological reserve and increased vulnerability to detrimental health outcomes,^1^ manifesting as poor health status and reduced recovery from insults such as infections and surgery.^2^ Frailty is associated with a five-fold increased risk of transitioning to residential care and a two-fold higher risk of falls, fractures and mortality.^3^

The aetiology of frailty remains unclear and is likely heterogeneous. Large, epidemiological studies linked frailty to genetics,^4^ co-morbidities and lifestyle factors, notably smoking, low levels of physical activity and poor nutrition.^5^ Molecular and physiological approaches are urgently needed to gain mechanistic insights into frailty pathophysiology. Recent reviews identified inflammation, gut microbiota and amino acid metabolism as key systems,^6^,^8^ with epigenetic alterations^9^ and the hormonal environment^10^ playing mediating roles. Despite the potential for longitudinal discovery of frailty biomarkers,^11^,^13^ many are either non-specific (inflammation) or relate to single aspects of frailty (muscle function) and lack longitudinal data.^14 15^

Recent longitudinal studies show improvement in frailty measures in most individuals following kidney transplantation,^16^,^18^ though mechanisms remain unclear. Studies consistently report significant reductions in physical frailty 12 months post-transplant, despite an initial increase caused by surgical stress.^19^ These findings indicate that kidney transplantation substantially improves frailty, demonstrating a marked reduction in its severity and underscoring the procedure’s positive impact over time.

A scoping review of frailty measurement in solid organ transplantation found that of 101 included studies, 48.8% of studies did not use an established frailty tool, and the Fried Frailty Phenotype was most common (44.7%).^20^ The Frailty Index (FI) assesses multiple health domains, including physical performance, cognitive function, nutritional status and psychosocial factors, comprehensively capturing the multifaceted nature of transplantation. A FI integrates physical aspects such as peripheral muscle strength and mobility, and also underlying determinants such as cognitive impairment, oral deficits and loss of independence. By addressing these interconnected factors, this measure identifies a wider range of vulnerabilities, offering a fuller picture of overall health status and potential risks.

This study aims to characterise changes in frailty status following kidney transplantation, compared with patients who remain on dialysis awaiting a transplant, as well as older people at baseline and follow-up. To gain a better understanding of frailty aetiology, this study will explore associations between frailty status and endocrine status, the metabolome, the microbiome, markers of microcirculation and tissue integrity.

It is hypothesised that frailty secondary to kidney failure and age-related frailty may be associated with similar physiological measures. These measures are anticipated to improve in most transplant recipients (as frailty typically improves,^16^ though individual trajectories will vary). Similarly, measures may worsen in patients remaining on dialysis or with age-related frailty, though responses will be heterogeneous.

The research questions for this study are:

- What are the longitudinal changes in frailty status (as measured by the FI) in each group?
- What is the association between frailty (as measured by the FI) and the molecular and physiological measures at baseline?
- How do the molecular and physiological measures co-vary with the change in frailty status over the 12-month study period?
- Are baseline (Time 0) molecular and physiological measures associated with frailty at 12-month follow-up?
- How do molecular and physiological measures change after transplant?

## Methods

### Study design

This longitudinal study will recruit 150 patients: Group A – adult patients with kidney failure receiving transplant (aged ≥40 years; n=50), Group B – adults with kidney failure active on the waiting list (aged ≥40 years; n=50) and Group C – older community-dwelling adults without kidney failure (aged ≥65 years, or ≥55 years for Aboriginal and Torres Strait Islander patients; n=50).

**Figure 1:**
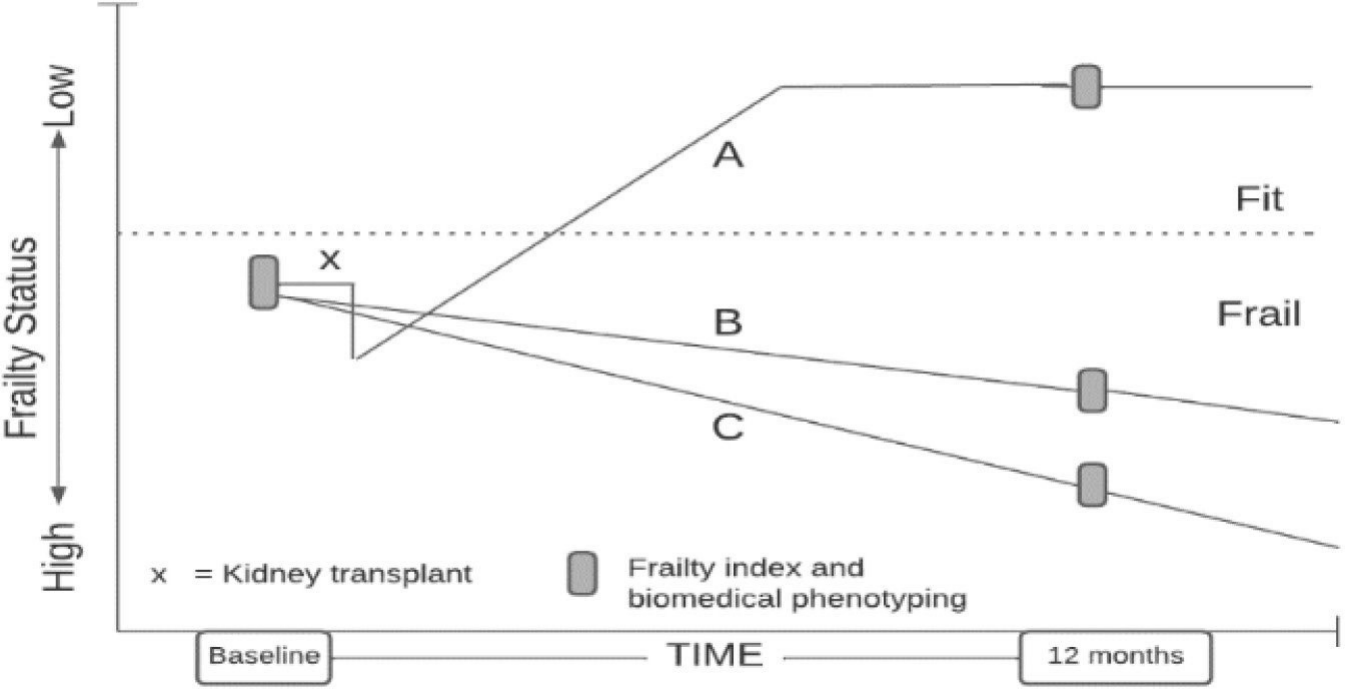
Study cohorts and anticipated trajectories. This figure illustrates the hypothesised mean changes in frailty status over time for the three study cohorts (Group A – kidney transplant recipients (anticipated improvement), Group B – kidney failure not receiving a transplant (anticipated stable or worsening) and Group C – older, community dwelling participants without kidney disease (anticipated slight decline)). The plotted trajectories represent anticipated mean changes based on findings in the published literature.^16^,^1863^ While these slopes show the expected group averages, they do not represent individual patient trajectories (ie, improvement, no change or worsening). Actual, empirically derived trajectories and their CIs will be reported in the study results.

Group C comprises older, community-dwelling adults who, based on previous studies^16 21 22^ and clinical frailty scales (CFSs) of 2–6, are anticipated to have similar baseline FI scores to Groups A and B, enabling comparison of age-related frailty with disease-related frailty (figure 1).

Recruitment commenced in January 2024, continuing 18 months until July 2025, with final 12-month follow-up assessments completed by July 2026. Measures are collected at baseline and at 12 months.

### Study outcomes

In each stream, data will be paired over time points. The primary outcome, FI as measured by the FI-Short Form (FI-SF) will be examined in relation to study measures including clinical metadata (age, sex, ethnicity, body mass index (BMI), medications, comorbidities), immune parameters (IL-6, C Reactive Protein (CRP)), kidney function (creatinine, blood urea nitrogen, estimated glomerular filtration rate (eGFR), electrolytes), nutrition intake, endocrine markers (cortisol, dehydroepiandrosterone (DHEA), sex hormones, insulin-like growth factor-1 (IGF-1), parathyroid hormone), serum metabolome, faecal microbiome (diversity metrics, taxonomic composition, functional metabolic capacity), circulating cell-free DNA (cfDNA) and mitochondrial DNA (mtDNA) and microcirculatory function (vessel density, flow indices, glycocalyx markers) (figure 1).^23^ Secondary outcomes include changes in FI over time in relation to the aforementioned measures, as well as baselined FI in relation to measures.

### Study population and setting

Participants will be recruited at the Princess Alexandra Hospital (PAH) – a large tertiary hospital in Brisbane, Queensland. Groups A and B will be adult patients (≥40 years) attending the PAH’s Queensland Kidney Transplant Service (QKTS). The service assesses all potential kidney transplant candidates and performs all kidney transplants for Queensland and northern New South Wales. Group C will comprise community-dwelling older people (aged ≥65 years, or ≥55 years for Aboriginal and Torres Strait Islanders) attending PAH Geriatric and General Medicine outpatient clinics.

### Eligibility

#### Inclusion criteria

Groups A and B – Kidney failure patients on the QKTS waitlist:

- Kidney failure (chronic kidney disease (CKD) stage 5 on dialysis, or pre-dialysis if pre-emptive living kidney donor recipient).
- Aged ≥40 years – chosen due to the increasing prevalence of frailty in kidney disease populations from this age.^24 25^
- Group A: patients anticipated to progress to transplant within 3 months of enrolment based on blood group compatibility, position on waitlist and clinical assessment.
- Group B: will be patients on the active transplant waiting list who are unlikely to receive transplantation within the next 12 months based on blood group and waiting time; patients may either be undertaking peritoneal dialysis or haemodialysis as their indicated renal replacement therapy.

Frailty is not a criterion for inclusion/exclusion for Groups A and B because frailty is highly prevalent in kidney transplant candidates (affecting 40%–70% depending on the measure used),^20^ and excluding non-frail patients would limit generalisability and our ability to examine the full spectrum of frailty changes.

Group A includes both patients receiving pre-emptive, live donor transplants and those on dialysis receiving either live or deceased donor transplants, as both pathways are common in clinical practice and both populations experience frailty. In contrast, Group B is restricted to patients on dialysis, as renal replacement therapy is a requirement for waitlist eligibility.

Group C – frail, older community-dwellers:

- CFS of 2–6. Our recruitment goal is to capture a cohort of older people with a similar frailty status to Groups A and B. In our study of 147 patients with kidney failure attending the QKTAC, mean FI was 0.23 (SD 0.10).^26^ Australian community-dwelling older adults requiring geriatric assessment and support have a CFS between 2 and 6, with mean FI 0.23.^27^
- Aged ≥65 years, or ≥55 years for Aboriginal and Torres Strait Island patients. For Group C, ≥65 years reflects the standard age threshold for geriatric assessment in Australia,^28^ while ≥55 years for Aboriginal and Torres Strait Islander patients recognises the well-documented earlier onset of age-related conditions in these populations, consistent with Australian clinical guidelines.^29^

#### Exclusion criteria

Groups A and B – Kidney failure patients on the QKTS waitlist:

- Patients who have or are planned to receive a transplant of any organ other than the kidney.

Group C – older community-dwellers:

- Life expectancy estimated to be <12 months.
- Advanced kidney disease (CKD stage 4 or 5).
- Individuals with a current or previous diagnosis of cancer, except for non-melanoma skin carcinomas that have been fully excised (eg, basal cell carcinoma or squamous cell carcinoma).
- Recent major surgery (<6 months).
- Major organ failure.
- Inability to provide written informed consent.

### Recruitment

Over 18 months, ~480 people will be assessed by the QKTS clinic for transplant feasibility. Previous work from Weerasekera *et al* suggests that FI assessment was feasibly completed in 62.8% of eligible patients (147/234).^26^ Using the expertise of transplant clinicians, we aim to recruit 50 patients anticipated to progress to transplant within 3 months (recruited to group A), and a target of 50 people who are unlikely to proceed to transplantation in the coming 12 months (Group B). Older people without kidney failure attending PAH outpatient clinics (n=50) will be recruited for Group C. Recruitment will be conducted over 18 months.

### Sample size calculation

For the younger groups with kidney failure (Groups A and B), previous data suggests that the mean age will be 52 years and mean FI will be 0.23.^26^ Based on significant reductions in physical aspects of frailty post-transplant, the FI in Group A is expected to reduce from mean=0.23 at baseline to the age-norm FI mean=0.06 at 12-month follow-up.^16 21^ This represents a scenario assuming substantial frailty improvement. If actual improvement is more modest (eg, from 0.23 to 0.15), power for between-group comparisons would be reduced. However, our primary analysis focuses on within-group changes using paired analyses, which have greater statistical power than between-group comparisons. Additionally, we would retain >80% power to detect a more conservative between-group difference of 0.08 FI units. The frailty trajectory in patients with kidney failure who do not receive a transplant is currently unknown. For Group C using the CFS to screen frailty, mean FI is expected to be 0.23 at baseline and increase by 3% each year, meaning that at 12 months, mean FI is expected to be 0.237.^30^ Therefore, there is sufficient power (>95%) to test the difference in means at follow-up between Group A and Group C, based on an independent samples t-test, with α set at 5% (two sides) and σ=0.10.

Multiple testing corrections will be applied given the high dimensionality of omics data and the potential for false positives. Independent data axes will be identified using principal component analysis (PCA) to control for false discovery rates (FDRs), with the denominator for multiple testing correction determined by the number of principal components explaining 99% of the variance. PCA requires complete data across all variables; missing data will be handled through appropriate imputation methods or by conducting PCA on complete cases with sensitivity analyses to assess the impact of missing data patterns.

### Data collection

All participants who consent to participation will be allocated a consecutive ‘participant identification (ID) number’ which will be used throughout data collection and analysis.

We acknowledge the logistical challenges of baseline data collection immediately before deceased donor transplantation. However, this timing is essential for capturing true baseline frailty status pre-transplantation. Where feasible, data will be captured retrospectively as close to the event as possible (surveys, stool sample <48 hours from transplant).

To facilitate dimension-reduction analyses such as PCA, we will prioritise collection of core measures at each time point for all participants. Data collection includes quality checks to minimise missing data, with research staff trained to identify and address incomplete assessments during study visits.

Group A:

1. Patients on the transplant waiting list or being worked up for a pre-emptive living kidney donor transplant will be mailed information about the study and informed that they may be asked to participate within the next 18 months (ReFIT pre-emptive letter).
2. If they meet inclusion criteria and present for a deceased donor transplant, participants will be consented by research staff just before transplantation. At that time, the blood will be drawn, and a research nurse will complete the FI and Australian eating survey (AES), while another member of the research team will complete the oral microcirculation. If possible, stool sample collection kits will be provided and collected before the transplant. For this group, it is not feasible to collect oral microcirculation, blood or stool samples post-transplantation due to the acute changes in haemodynamics,^31^ as well as the provision of medications that will influence microbiome measures.^32^ For Group A deceased donor recipients on dialysis, blood samples will be drawn prior to transplant surgery during preoperative procedures, but stool sample collection and oral microcirculation may not be guaranteed.
3. Patients receiving a transplant from a live donor have surgery scheduled ahead of time and so will have their FI assessment and AES conducted at the pre-transplant review (~1 week before transplant). At this time, they will be given their stool sample collection kit, which they can return by post using the prepaid and addressed package or return in person on the day of transplantation. On the day of transplantation, the blood will be drawn, and oral microcirculation will be taken before the transplant.
4. Transplant patients are scheduled to have a follow-up assessment completed around their 12-month post-transplant review or at a separate return visit. All measures indicated in table 1 will be taken at this time point except for the stool sample that will be provided on the visit and asked to be mailed via post using the prepaid and addressed package.

**Table 1:** Defines and describes measures taken at each time point to explore outcomes

Measure	Description
Questionnaires	
Frailty index	A frailty measure using an accumulation of deficits approach across different domains, including cognition, function, sensorium, mood, continence, nutrition and co-morbidities. Conceptualised and validated by Rockwood and Mitnitski, it has been subsequently validated throughout several cohorts worldwide.^5 41 57 58^
Australian eating survey	The Australian eating survey is a 120-item food frequency questionnaire with 15 supplementary questions. It is designed to collect information about participants’ dietary intake over the previous 3 to 6 months. It is semi-quantitative with a standard portion size provided for each food item and determined using natural serving size where possible. It has been thoroughly evaluated for reliability in Australian children and adults and has shown to be valid against biomarkers and food records.^53 59 60^
Blood test	
Health indicators	Circulating inflammatory markers (IL-6, CRP), kidney function blood tests: serum creatinine, eGFR, urea, albumin.
Molecular and physiological indicators	Endocrine status (cortisol, DHEA, oestradiol, progesterone, testosterone, SHBG, IGF-1, parathormone), metabolome (amino acids, lipids, organic acids, acylcarnitines, bile acids, SCFAs), circulating cell-free DNA and mitochondrial DNA, measures of the microcirculatory and endothelial health (thrombomodulin, syndecan-1, ADMA, actin, gelsolin, gc-globulin, DNase-1).
Tissue integrity	Cell-free DNA (cfDNA), mitochondrial DNA (mtDNA) and methylation status. cfDNA and mt DNA are emerging biomarkers of cellular stress, tissue damage and biological ageing. Elevated cfDNA and mtDNA have been associated with frailty and mortality in older adults and may reflect accelerated ageing processes in kidney disease.
Microvascular health assessment	
Oral microcirculation side stream dark field videomicroscopy	A non-invasive imaging tool that assesses the microcirculation through quantifying physiological parameters such as capillary density, microvascular flow index, perfused vessel density, proportion of perfused vessels and microcirculatory heterogeneity index. Green light-emitting diodes from the sidestream dark field camera are absorbed by haemoglobin in erythrocytes to allow for their visualisation.^61^ It has been validated in the clinical setting for critically ill patients subject to trauma and intensive surgery^62^ and has been successfully applied to kidney failure patients and associated with frailty.^63^ Endothelial dysfunction is associated with glycocalyceal shedding (evaluated by circulating syndecan-1 and thrombomodulin) and the inhibition of nitric oxide by the circulating endogenous inhibitor, asymmetric dimethylarginine (ADMA).
Provided at the time point and mailed back	
At-home gut microbiome sampling kit	Participants are provided with a gut microbiome sampling kit (Microba Insight) to take home to collect a sample of their stool microbiomes that can be returned by express post. The sample is stabilised and can sufficiently be preserved at ambient temperatures for periods of up to 4 weeks,^64^ by which it will then be stored at −80°C until mass sequencing occurs.
Bristol stool chart	Within the kit will be a piece of paper that allows participants to write the Bristol Stool classification of the stool that will be requested to be filled out and mailed back with the stool sample. The Microba Insight kit provides participants with a visual reference of each stool type.

DHEA, dehydroepiandrosterone; IGF-1, insulin-like growth factor-1; SCFAs, Short-Chain Fatty Acids; SHBG, sex-hormone binding globulin.

Group B:

1. Patients on the transplant waiting list will be mailed information about the study and informed that they may be asked to participate within the next 18-month period (ReFIT pre-emptive letter).
2. Patients identified by a nephrologist as unlikely to receive a transplant in the next 12 months will be approached about participation in the study.
3. Patients identified as suitable participants in this group will be receiving a form of dialysis treatment and will have regular contact with the PAH Nephrology team, and opportunities will be provided to be contacted by those involved with the study.
4. Non-transplant patients on dialysis have regular contact with PAH Nephrology, allowing for the completion of the FI, AES, oral microcirculation and for blood to be drawn on the same day of a routine dialysis visit, prior to dialysis. This will be completed at their first session after consent and 12 months later.

Group C:

1. Community-dwelling older adults who attend PAH outpatient clinics will be identified by outpatient clinic staff at routine consultations and will be provided with the study flyer before being asked if they agree to speak to a researcher. A researcher will then discuss the ReFIT study with the potential participants and provide them with a participant information sheet. For anyone who indicates they would like to participate and agrees to be contacted, a follow-up phone call a few days later will be organised to confirm their interest. This will allow potential participants time to consider and speak to others about participation. If they confirm interest when phoned, a researcher will organise a time for them to return to the PAH Clinical Research Facility (CRF) for a study visit which will involve consent and data collection.
2. Written, informed consent is required for study inclusion and must be provided before data collection.
3. After consent, screening will be conducted including confirmation of the inclusion criteria (age and CFS) and exclusion criteria (medical history).
4. For baseline measures, the FI will be completed, blood drawn, AES completed and oral microcirculation videomicroscopy recorded.
5. The stool collection kit will be provided to the patient with a prepaid and addressed package for return via post.
6. At the review that is scheduled for 12 months, follow-up FI assessment, blood sample, dietary and stool questionnaires and oral microcirculation videomicroscopy will be collected. Like the baseline measures, this will be conducted at the PAH CRF.

### Informed consent

Patient information and consent forms (PICFs) have been developed in collaboration with consumer stakeholders to ensure high readability, inclusivity and a consumer-centric approach. Research nurses will obtain consent and those who are interested will be provided with the opportunity to discuss the implications of participation. It will be explained that participation is voluntary with no bearing on medical treatment.

As mentioned above, participant ability to provide informed consent may vary between groups. For Group A, informed consent from each participant will be sought as early as possible after surgery is scheduled, while participants in Group B or C can be provided additional, relevant information with ample opportunity to ask questions at clinic visits. Living donors consent independently to the QKTS, where their informed consent meets standards introduced by the Australia and New Zealand Paired Kidney Exchange Programme.^33^ After these discussions, participants will have a reasonable amount of time to enquire further and decide on participation.

### Patient and public involvement

Consumer stakeholders were involved in the development of PICFs and recruitment flyers to ensure high readability, inclusivity and a consumer-centric approach. Consumer representative members of the Australian Frailty Network were further recruited for ongoing participation in Study Steering Committee meetings, ensuring constant input from consumer stakeholders. Consumers will interpret the results to ensure that the findings, when disseminated via newsletters and email summaries, are written in accessible language, reflect real-world perspectives and lead to meaningful conclusions.

### Organ donation procedures

Organ donation abides by the state,^34^ national^35 36^ and international^37^ guidelines, as well as professional guidelines^38^ and legal frameworks to ensure all parties, including donors, are protected from exploitation during the process. The overall process is aligned with community expectations that altruistic donations of human cells, tissues and organs are treated respectfully, shared equitably and used effectively for the benefit of all. Care for live organ donors begins with rigorous medical, psychological and social assessment to determine suitability prior to donation.^39^ To ensure impartiality, potential donors are referred to a specific clinic within the PAH to undergo extensive medical, surgical, psychological and social work-based assessment before being considered. Potential donors are provided with extensive time to consider the advice, particularly around their kidney health. Postdischarge, donors are followed up in the clinic to ensure complete recovery and any issues are re-referred to the QKTS. Living donors are also eligible for financial support during their recovery through the Australian Government’s Supporting Living Donors Programme, where financial reimbursement is available for up to 18 weeks of paid leave, once-off payments to help with out-of-pocket costs, and for travel and accommodation.^40^

### Measures

Table 1: overview of measures taken at each study time point. This table details the measures used within the study, categorised by the type of assessment. These measures include questionnaires (Frailty Index and Australian Eating Survey) to assess cumulative deficits and dietary intake, respectively; blood tests measuring various health indicators, molecular and physiological indicators and markers of tissue integrity; a microvascular health assessment using non-invasive oral microcirculation sidestream dark field (SDF) videomicroscopy and provided at the time point and Mailed Back kits for at home gut microbiome sampling and Bristol Stool Chart classification. The table provides the measure’s name and a brief description, including the conceptual basis, collection method or key assessed parameters, along with relevant citations supporting their validation and use.

#### Demographic/health indicators

Demographic and health information will include age, sex, ethnicity, smoking status, BMI, sitting blood pressure, current medications, exercise (type and duration within the prior 24 hours) and time fasted. For Groups A and B, additional renal-specific indicators will be captured including current dialysis protocol (type and time since last dialysis), previous transplant history and kidney function blood test data. This data is collected routinely at dialysis and clinic appointments and stored on Queensland Health’s electronic medical record system, ieMR. For Group C, a blood sample will be sent to Pathology Queensland to analyse kidney function parameters, where tests quantify serum levels of sodium, potassium, chloride, bicarbonate, creatinine and urea. Participant responses to questions will be entered into the REDCap database in a re-identifiable manner. All clinical employees of Metro South Health (the governing body of the PAH) may be involved in data collection from ieMR.

#### Frailty index

The FI-SF will be completed via interview and will take approximately 10 min.^22^ The FI-SF is a 58-item (deemed a ‘deficit’ if present for an individual) survey that has been based on the original FI by Rockwood *et al* at Dalhousie University.^41^ It captures domains relevant to Comprehensive Geriatric Assessment and represents multiple health domains such as medical conditions, function, peripheral muscle strength, Activities of Daily Living, nutrition and cognition.^42^ The FI-SF has been validated in previous work from our group in several populations, specifically in kidney failure outpatients attending QKTS assessment clinics.^22 43^ After the interview, the total deficits are summed and subsequently divided by the number of potential deficits to give an FI. For example, if a participant has 10 deficits from the total 58 deficits, they have an FI of 0.17.

The FI was selected as the primary outcome measure for several reasons. First, it has demonstrated responsiveness to change in longitudinal studies across several age-related and chronic disease-related populations.^44^,^46^ Second, unlike phenotypic measures (eg, Fried Frailty Phenotype) that focus primarily on physical frailty, the FI captures multiple health domains including cognitive function, nutrition, mood and comorbidities, providing comprehensive assessment relevant to the multisystem challenges expected with transplantation. Third, the FI has been validated in kidney failure patients attending transplant clinics in our previous work.^22^ Finally, the accumulation of deficits approach aligns with the conceptual framework that frailty represents multisystem decline, which we aim to characterise through molecular and physiological measures.

#### Sample collection

Whole blood from participants will be collected in two cfDNA collection tubes (PAXgene 10 mL Blood cfDNA tube) and two edetic acid (EDTA) tubes (BD Vacutainer EDTA 10 mL tube) for downstream analyses described below. EDTA samples are spun on site while cfDNA collection tubes will be couriered on the same day of collection to the Institute for Molecular Bioscience (IMB) at UQ.

#### Endocrine status

The internal hormonal environment will be assessed through liquid chromatography-mass spectrometry (LC-MS/MS) of plasma samples from the whole blood EDTA tubes. This will quantify the changes in cortisol, DHEA, oestradiol, progesterone, testosterone, sex-hormone binding globulin, IGF-1 and parathormone between baseline and 12 months. To enable absolute quantitation of these analytes, individual analytical standards (along with deuterated compounds) may be used for developing a targeted quantitative mass spectrometry method. The samples will be extracted by solid-phase extraction kits or organic solvent precipitation for analysis using Shimadzu 8050 QQQ and/or ABSCIEX QTRAP – 5500 LC-MS/MS instrument. This will be based on the analytical requirements for the development of the assay. A targeted LC-MS approach allows for the quantification of hormonal levels, which affect the pro-inflammatory state within multiple tissues, implicated in both the development of age-related frailty^10^ and frailty in kidney disease.^47^

#### Metabolomics

The sample analysis is generally performed using liquid and/or gas chromatography and mass spectrometry instruments to capture all possible classes of circulating metabolites. The metabolites of interest could be initially identified using untargeted LC-MS/MS and/or gas chromatography-mass spectrometry (GC-MS) methods. After metabolites of interest are identified, a validated, targeted metabolomics panel can be used for absolute quantification. Prior studies have used an untargeted metabolomics approach, which has identified several metabolites and their associated pathways linked to frailty, including major amino acids, biogenic amines and metabolites, oxidative stress markers, vitamins, acylcarnitines, bile acids, Krebs cycle metabolites, short-chain fatty acids and reactive carbonyl compounds (28). All metabolites have the potential to be quantified in a targeted panel along with any novel metabolites indicated in the untargeted panel. Plasma samples will be processed using organic solvent extraction as per existing protocols^48 49^ and analysed on either LC-MS/MS, GC-MS or both.

#### Circulating cfDNA and mtDNA

Plasma for cfDNA extraction will be carefully separated with two centrifugations (PAXgene cfDNA tube, followed by FALCON Conical Centrifuge Tube) to avoid post-collection lysis of blood lymphocytes that can artificially increase cfDNA, before being stored at −80°C. For subsequent cfDNA extractions, plasma samples will be thawed at room temperature, and extraction of cfDNA will be carried out for all samples using the same protocol. This will be using the QIAamp Circulating Nucleic Acid Kit (catalogue #55114; QIAGEN) or similar technology (that will maximise cfDNA yield). DNA will then be eluted in a small volume to maintain high concentrations (ie, 25 µL-50µL of reagent).

To quantitate and size total cfDNA fragments, each sample will be run on a high-sensitivity chip (Agilent 2100 Bioanalyser) or cfDNA ScreenTape (Agilent ‘Tape-station’). If concentrations are at least 5–10 ng/mL we will proceed with methylation capture screening to deconvolve the sample into cells/tissues of origin. For mt-cfDNA, target sequences from the mitochondrial genome and nuclear genome will be used to target respective regions of the DNA using real-time PCR (RT-qPCR, ViiA 7 System, Applied Biosciences). Samples will be tested in duplicate with a standard curve using control gDNA and/or synthetic DNA (gblock, IDT) for copy number quantitation.

#### Microcirculation and tissue integrity

Oral microcirculation SDF video microscopy will be used to assess flow and vessel density in the microcirculation, captured on the mucosa of the participant’s sublingual area. AVA V.4.3 software (MicroVision Medical, Amsterdam, the Netherlands) is used to take real-time video clips of the sublingual microcirculation, where semi-automated analysis of clips can determine total vessel density, perfused vessel density, proportion of perfused vessels, microcirculatory flow index and microcirculatory heterogeneity index. The use of semiautomated analysis software is preferred over the updated, fully automated software due to the significant differences between numeric results, affecting the overall agreement of data obtained by the fully automated software.^50^ Therefore, expert judgement and experience will influence this analysis. Although variable, the mean duration of SDF video microscopy measurement under ‘field conditions’ is 5:32±2:22 min for the recording of a sufficient quality video to be analysed in a prehospital, emergency setting.^51^ Since our study environment allows for multiple measures to be taken, in a non-acute setting, we estimate that this measurement should take approximately 15 min to complete.

Circulating levels of thrombomodulin, syndecan-1, asymmetric dimethylarginine, actin, gelsolin, gc-globulin (vitamin D binding protein) and DNase-1 will be measured by ELISA (Abcam, Melbourne, Australia) and/or by LC-MS.

#### Microbiome

Participants will be provided with a Microba Insight sampling kit, an at-home stool collection kit, at their pre-transplant review, before transplantation, during routine dialysis or a visit to the CRF – depending on group enrolment and timing of transplant. All participants will be provided with these kits at both baseline and 12-month time points, with prepaid and return-addressed packages.

The microbial diversities, taxonomic identification and functional capacity of faecal microbiota will be measured through shotgun metagenomic sequencing (MGS). For MGS, libraries will be constructed using the Illumina Nextera XT DNA Library Preparation Kit (CA, USA) and with the Illumina NextSeq500 platform as 150 bp, paired-end reads to a sequencing depth of 3Gbp per sample; where data are generated from the extracted DNA from collected stool samples. A standardised methodology for lysis of microbial biomass, DNA clean-up and purification will be used.^52^ The purified DNA will then be fractionated before being assembled by contiguous sequences so that a library can be constructed and contaminating host DNA can be trimmed for a viable dataset to be produced. Data analysis software packages (eg, HUMANn3 or equivalent) will characterise functional and taxonomic attributes of the stool microbiota. Outputs include uni-/multivariate analysis for within (alpha-diversity) and between-sample (beta-diversity) measures of diversity.

The microbiome data will be complemented by key dietary and clinical metadata. The AES, a source of dietary metadata, is a semi-quantitative, 120-item questionnaire providing a breakdown of nutritional intake, quantifying macro- and micronutrients, fluids and phytochemicals from processing data in a pre-established database.^53^ It has been used across several settings and population groups as a dietary habit recollection tool for the past 3–6 months. It can be completed individually or with assistance and is completed in approximately 15 min. While this survey is likely to be completed during a QKTS visit, pre-transplantation, dialysis or CRF visit, the unpredictability of the deceased donor transplantation may mean that the AES will be completed retrospectively, postoperation on the ward. Stool consistency will also be measured as the Bristol Stool-Form Scale, a clinically validated measure that is also accepted within research contexts.^54^ Participants will indicate their self-rated stool consistency from which the faecal microbiota sample was taken.

### Material transfer

The two BD Vacutainer EDTA 10 mL tubes will be processed twice with a primary and secondary spin. The primary spin will centrifuge the BD Vacutainer EDTA Tubes 10 mL at 2500 g×15 min, at 4°C, and the subsequently produced plasma is pooled evenly into two 15 mL pre-labelled FALCON Conical Centrifuge Tubes. Pooling must be completed within 15 min of the primary spin. FALCON Conical Centrifuge Tubes are then processed again in a secondary spin for 2500 g for 15 min at 4°C. After this, the plasma is aliquoted into 1.5 mL pre-labelled, micro centrifugation Eppendorf tubes (SARSTEDT, SC001501) X 0.5 mL each. The Eppendorf tubes can be stored at −80°C at the PAH for a maximum of 72 hours after collection. They are to be collected by a research staff member to the UQ School of Biomedical Sciences, and IMB, UQ, St Lucia Campus, for further centrifugation, storage and analysis. Storage will occur in a −80°C freezer until analysis.

Once collected, the FLOQswab-ADT collection chamber within Microba Insight stool collection kits stabilises the sample until further processing. Faecal samples will be delivered to Microba at ambient temperature, before DNA extraction and sequencing.

Oral microcirculation video data will be captured at the clinic visits and stored on a UQ-owned laptop for analysis as pseudo-anonymised files. Following analysis, data will be stored on the UQ research data manager system.

### Safety considerations/risks

The research staff conducting all assessments (FI, AES and oral microcirculation) and blood tests will receive training on these measures before participant recruitment and data collection. The study’s associate investigators will also be available for contact where necessary, including regularly scheduled meetings with the investigator team. Data monitoring in the REDCap database will be conducted monthly by the data manager and project team to ensure the completeness of assessments. Records will also be locked by the delegate after they are completed so that accidental changes cannot be made. Only necessary staff members will have the ability to access REDCap records.

### Data analysis

Statistical analyses will focus primarily on the FI as the primary outcome measure, with exploratory analyses examining associations with secondary outcomes (clinical metadata, molecular and physiological measures).

Participant characteristics for the whole sample, and Groups A, B and C separately, will be reported. Comparisons between groups will be conducted using t-tests for normally distributed, continuous data, Mann-Whitney tests for non-normal continuous data and χ^2^ tests for categorical variables.

The FI will be analysed primarily as a continuous variable to maximise statistical power and capture subtle changes. In secondary analyses, we will stratify the FI using established thresholds (eg, FI≤0.10=non-frail, 0.10≤FI≤0.21=pre-frail; FI>0.21=frail) to facilitate clinical interpretation and comparison with existing literature.

To address the challenge of multiple hypothesis testing across high-dimensional omics data, we will implement the following strategies: (1) FDR control using the Benjamini-Hochberg procedure will be applied within each omics platform, (2) PCA will be used to identify independent data dimensions with the effective number of tests determined by the number of principal components explaining >99% of variance, (3) For exploratory analyses, we will report both uncorrected and FDR-corrected p values with appropriate caveats and (4) Key findings will be prioritised for validation in future studies. Baseline differences among groups will be addressed by including relevant covariates (age, sex, comorbidities, medications) in multivariate models and by reporting both adjusted and unadjusted analyses. We recognise that the sample size relative to the number of potential biomarkers limits our ability to conduct definitive hypothesis testing. This study is designed to be exploratory and hypothesis-generating, identifying promising biomarkers and pathways for focused investigation in larger, confirmatory studies.

#### Research question 1: what are the longitudinal changes in FI in each group?

Changes over time in Group A FI will be analysed using linear mixed models. Models will account for subject-to-subject variability via random effects, with time (baseline vs post-transplant) and demographic variables as fixed effects. Changes reported are based on marginal means. Baseline to 12-month FI changes in non-transplant and older groups will also be evaluated using linear mixed models, adjusted for relevant demographic and health fixed and random effects. Data will examine between-group differences using intention-to-treat methodology and complete cases/imputation models as sensitivity analyses.

For missing FI items, the denominator (variables assessed) will be decreased by missing items to a minimum of 30 variables—the minimum for valid FI assessment, or imputation techniques may be applied as appropriate. Medication or comorbidity data, contributing largely to FI deficits, can be obtained from chart reviews and ieMR. A participant would need 18 missing deficits for invalid FI, which we do not anticipate.

#### Research question 2: what is the association between the FI and molecular and physiological measures at baseline?

In each stream, data will be paired over time points and pre-/post-transplantation for Group A. Paired normalised data will be analysed by t-test and non-normalised data with Wilcoxon signed-rank test. Association strength of each measure with baseline FI will be explored with stepwise multivariate linear regression using backward elimination, retaining variables significant at p<0.1, as well as being adjusted for pre-specified covariates. Remaining predictors will be examined for multi-collinearity using variable inflation factors with appropriate adjustments, and variance percentage associated with each variable will be extrapolated.

#### Research question 3: what are the concurrent associations between changes in molecular and physiological measures and changes in frailty?

The transplant procedure is expected to affect both FI and molecular/physiological measures. Association between post-transplant changes in molecular/physiological measures and post-transplant FI changes will be explored using multivariate analyses to quantify relationships. Similar analyses will be undertaken for follow-up changes in non-transplant and frail older groups.

#### Research question 4: do baseline molecular and physiological measures predict subsequent changes in frailty?

Linear mixed models will be used to assess the association of baseline biomedical (or frailty) scores with change in frailty (or biomedical) measures at follow-up, adjusted for baseline (to control for regression to the mean), and relevant covariates determined by a backward elimination approach.

#### Research question 5: how do molecular and physiological measures change after transplant?

Principal components and/or factor analysis will quantify major axes of variation in molecular/physiological measures post-transplant, informing follow-up analyses and prioritising datasets for future exploration in larger studies. Combining FI with specific molecular/physiological measures to generate a ‘clinical-molecular and physiological frailty score’ could predict adverse outcomes in future. Preliminary analyses using model diagnostics (eg, AuROC, sensitivity, specificity) will identify optimal cut-off(s) and numbers needed to treat for broader analysis in subsequent studies.

### Data management

#### Data storage

Electronic data will be captured and stored in REDCap, a central, web-based database that is secure and password-protected. REDCap is stored on a server managed by the Queensland Clinical Trials and Biostatistics Centre at UQ, Australia. Data from REDCap will be backed up regularly and stored electronically in password-protected folders on a secure network drive on Australian servers.

Written information about the participants will be stored in a locked cabinet with access restricted to members of the research team. All electronic records containing personal information will be stored on a password-secured, firewall-protected network within Metro South Health. The information that is sent externally will only be identified using a coded format.

De-identified data about biomedical parameters (blood/stool analysis and microcirculatory video microscopy parameters) will be stored on the UQ Research Data Management System. Blood samples will be stored in a minus 80 degree Celsius freezer in its designated laboratory and plasma samples will be securely stored in the designated laboratories at UQ (SBMS/IMB). The stool samples will be stored at room temperature until analysis.

#### Data retention

After the closure of the trial, investigators will retain study documentation relating to ethics committee approvals, correspondence and re-identified (coded) REDCap data for a minimum of 15 years.

#### Data disposal

All data collected by the research staff approved by the human research ethics committee, that is identifiable, will be stored in patient files at the PAH and destroyed via MSH secure document destruction. After the minimum data retention period has lapsed, electronic files may be disposed of following usual practices at the site and as per Metro South Human Research Ethics Committee approval.

#### Destruction of biospecimens

After analysis has been performed, the samples will be kept until the completion of the study, in case during the final analysis of results with all participant samples, there are outlying results that indicate technical issues, and a re-assay should be performed. Per the QLD State Archives University Sector Retention and Disposal Schedule, and the Australian Code for the Responsible Conduct of Research, the destruction limit for biospecimens will be 5 years after the last action.^55 56^

Destruction of biospecimens will be performed as per UQ Policy and Procedure Library 2.40.15. Work areas and contaminated metals will be disinfected with ethanol (80% v/v) after the completion of the task, followed by a minimum of 20 min of exposure to UV light. Used tubes, plastic pipette tips, glassware and other contaminated items will be decontaminated using bleach (1% sodium hypochlorite concentration, as this is the active ingredient) for a minimum of 12 hours of contact time. The bleach will then be decanted, and the pipette tips/cell culture items or other contaminated materials will be discarded via the clinical waste stream or, if they are glassware, washed before autoclaving for cleaning. A note will be taken of the bleach expiry date before use. Bleach stock shelf life is 1 year, and the concentration of bleach will decrease with time. Information regarding this can be found on the bleach bottle. Appropriate personal protective equipment (back-fastening or side-fastening lab gown, gloves, covered footwear and safety glasses) will always be worn during work and waste disposal/decontamination.

For the stool samples collected and extracted from swabs, the raw sample is destroyed by the protocol of DNA extraction. Any residual DNA will be discarded according to standard protocols for biological specimens.

### Ethics and dissemination

This study will be conducted with all stipulations of this protocol, and the conditions of the ethics committee approval. Ethical principles have their origin in the Declaration of Helsinki, all Australian and local regulations and in the spirit of the standard of Good Clinical Practice (as defined by the International Conference on Harmonisation).

Ethics approval was received by the Metro South Health Research Ethics Committee (HREC/2023/QMS/95392) and ratified by the University of Queensland.

The ReFIT study has developed a plan for disseminating the results so that findings are shared through multiple content-specific channels. Targeted, high-impact, peer-reviewed journal publications and presentations at academic conferences will ensure scientific dissemination, while summaries will be shared with participants via newsletters and email summaries. Public outreach will be conducted through media releases and collaboration with relevant health organisations. All findings will be communicated in clear, accessible language, with an emphasis on the study’s implications for basic science and clinical practice.

## Discussion

The longitudinal, comparative nature of this protocol, which includes frailty status measurement at baseline and 12 months across three distinct cohorts (transplant candidates, waitlist patients and older community-dwelling adults), constitutes the major strength of the design. This methodology is expected to yield temporal insights into frailty that a cross-sectional study cannot. Specifically, the comparison between age-related and disease-accelerated frailty is expected to enable the identification of bio-mediators that are unique to kidney failure, suggesting targets for therapeutic intervention in younger patient populations. The longitudinal data on waitlist patients is anticipated to show which physiological measures are most predictive of decline over a clinically relevant period.

The inherent age difference between the patient groups and the control cohort represents a necessary design constraint to test the hypothesis of shared frailty mechanisms across the lifespan. However, this is also a methodological limitation that must be carefully addressed. We will include age as a covariate in all between-group and longitudinal statistical models to account for its independent effects on molecular and physiological measures. This statistical mitigation is critical for ensuring that the final data, our expected outcomes, allow us to isolate the specific effects of the disease state from the normal ageing process. The robustness of this statistical approach is vital for the eventual clinical relevance of our findings.

In summary, the design is structured to provide high-resolution, time-dependent data on frailty. The expected outcomes are the reporting of molecular and physiological measures associated with frailty secondary to organ failure and/or with age-related frailty. This will not constitute causal proof of frailty, but rather form the basis for future, hypothesis-driven exploratory studies.
