# Supplementary material for: ReFIT study (reversing frailty in transplantation): protocol for a longitudinal study to assess clinical and biomedical changes in frailty through kidney transplantation
Source: BMJ Open. 2026 Mar 2;16(3):e100158. doi: 10.1136/bmjopen-2025-100158 (PMC12958976; doi:10.1136/bmjopen-2025-100158)
Supplement: Supplementary file 1 [file bmjopen-2025-100158.reviewer_comments.pdf]

PEER REVIEW HISTORY

BMJ Open publishes all reviews undertaken for accepted manuscripts. Reviewers are asked to complete a checklist review form and are provided with free text boxes to elaborate on their assessment. These free text comments are reproduced below.

ARTICLE DETAILS

Title (Provisional)

The ReFIT Study (Reversing Frailty In Transplantation): Protocol for a Longitudinal Study to Assess Clinical and Biomedical Changes in Frailty Through Kidney Transplantation

Authors

Payne, Trent; Shaw, Alyra; Hanjani, Leila Shafiee; Homes, Ryan; Giddens, Fiona; Ravuri, Halley Gora; Yap, Chloe X.; Walsh, James; Kumar, Vinod; Garton, Fleur C; Rhee, Handoo; Huang, Alan; Francis, Ross S; Reid, Natasha; McAdams-DeMarco, Mara; Gordon, Emily; Midwinter, Mark; Hubbard, Ruth

---

VERSION 1 - REVIEW

**Reviewer**	**1**
**Name**	**Lorenz, Elizabeth**
**Affiliation**	**Baylor College of Medicine**
**Date**	**28-May-2025**
**COI**	**None**

Comments to the Authors:

Abstract:

-- Group A is called group BA. Please correct.

-- Clarify that participants in Groups A and B will be ≥ age 40.

-- This nonrandomized study will determine associations, not causality or pathophysiology. Recommend rephrasing sections of the manuscript stating that causality or pathophysiology will be determined. This also applies to Introduction and Discussion sections as well.

-- Add brief description of planned statistical analyses.

-- Given that sample size was calculated using Frailty Index, consider identifying Frailty Index as primary outcome and other outcomes as secondary outcomes instead of co-primary outcomes.

Introduction:

-- What is the difference between the third and fourth research questions on page 10? They appear to be very similar.

-- The authors state that frailty will improve in patients undergoing kidney transplantation (group A) and worsen in groups B/C. This is not necessarily true. Studies have shown that patients some patents experience worsening frailty during the first year after kidney transplantation, and in many patients (including those in groups B/C), frailty status may not change during a 12-month period.

-- Outcomes are collectively referred to by different terms (e.g., ‘molecular and physiological measures’ vs. ‘biomedical measures’). Consider using the same phrase throughout the Introduction and the rest of the manuscript for consistency.

Methods:

-- Study Design: When outlining different groups, add age cut-offs for each group. Why does it say that Group C will be ‘frailty-matched’? If so, please clarify how this matching will be performed in the Methods.

-- Eligibility: Why was the age limit of ≥ 40 years chosen for groups A and B? Why was a lower age limit of ≥ 55 years for Aboriginal and Torres Strait Islanders chosen for group C? Please justify. Consider including ‘anticipated to progress to transplant within 3 months’ as eligibility criteria for group A.

-- Eligibility: Explain why frailty is not an eligibility criterion for groups A and B while it is in Group C?

-- Eligibility: Explain why Group A includes patients not on dialysis while Group B only includes patients on dialysis.

-- Sample Size Calculation: Was participant withdrawal planned for? If not, may need to increase enrollment rate to end up with 50 participants in each group upon study completion.

-- Data Collection: Please clarify whether samples will be taken prior to dialysis on dialysis days in Group A participants.

-- Measures: Please justify choice of Rockwood Frailty Index as primary outcome compared to other frailty measures. Is it responsiveness to change? I assume the majority of comorbidities assessed via the Frailty Index will not change during a 12-month period, except for renal failure which will automatically change in the kidney transplant group. Consider adding Frailty Index questions as supplementary material.

-- Measures: Provide rationale for each study outcome. For example, why include cell free DNA as a secondary outcome?

-- Data analysis: The study includes a large number of outcomes and few patients. Describe how you will account for multiple hypothesis testing and baseline differences among groups to minimize confounding and model overfitting.

-- Data analysis: Please mention primary and secondary outcome measures.

-- Data analysis: Aren’t research questions 3 and 4 essentially the same?

-- Data analysis: Is the Frailty Index score only going to be analyzed as a continuous variable? If not, include what cut-off will be used to define patients as frail for the analyses.

Table 1: Consider moving list of study measures outlined in Table 1 from Study Design section of the Methods to Measures section of the Methods. Include complete list of all measures in Table 1, not just examples.

Figures:

-- Figure 1: Studies have shown that patients some patents experience worsening frailty during the first year after kidney transplantation, and in many patients (including those in groups B/C), frailty status may not change during a 12-month period. Consider changing to study flow figure and focusing on study flow rather than hypothesized frailty trajectories across groups.

Reviewer 2

Name Bauer, Andrea Carla

Affiliation Universidade Federal do Rio Grande do Sul, Internal Medicine/ Nephrology

Date 17-Jun-2025

COI None

This protocol describes a longitudinal study examining changes in frailty following kidney transplantation, comparing three groups: transplant recipients, dialysis patients, and older, frail community dwellers. The core concept is potentially very valuable for understanding the pathophysiology of frailty. However, several practical and methodological concerns emerged during the review that need to be addressed before publication.

Pre-transplant Data Collection Problem: The biggest concern is the plan to collect baseline data "just before transplantation" for deceased donor recipients. This presents significant logistical challenges that may compromise the quality of the data. When patients receive notification of organ availability, they and their families are focused on getting to surgery, not participating in research assessments. The protocol requires blood draws, detailed questionnaires, microcirculation studies, and stool samples from individuals about to undergo major surgery. So, this timing may not capture the actual "baseline" frailty status.
Consider recruiting patients during their initial evaluation for transplant instead. While this approach would lose some who never receive a transplant, it would yield much better baseline data.

Statistical Analysis Needs Strengthening: While the authors acknowledge the "curse of dimensionality," the proposed solution appears inadequate. Principal component analysis for controlling false discovery rates across multiple omics platforms feels insufficient for the scope of this study. With 50 patients per group and potentially thousands of biomarkers, a serious multiple testing problem will arise, requiring a more sophisticated approach.
Additionally, the sample size calculation assumes frailty will improve from 0.23 to 0.06 post-transplant. This represents a significant improvement, which may be overly optimistic based on existing literature. Most published studies show more modest improvements. This assumption could leave the study underpowered for its primary

endpoint.

Group Matching Strategy Lacks Clarity: The manuscript repeatedly refers to "frailty-matched" groups, but the actual matching strategy remains unclear. Group C uses the Clinical Frailty Scale, specifically items 2-6, which includes people who are essentially well (CFS 2). For studying frail populations, including non-frail controls seems counterproductive. The age difference between groups (≥40 vs.≥65) also complicates the matching strategy and requires better justification.

Missing Measurements Post-Transplant: The inability to collect microcirculation and microbiome studies immediately post-transplant due to immunosuppression and hemodynamic changes creates data asymmetry that limits between-group comparisons. Consider adding an earlier post-transplant time point (perhaps 6 months) for these measures to address this limitation.

Frailty Index Application: The Frailty Index was developed and validated in older adults. The manuscript requires a more detailed discussion of how this tool will be adapted for the younger kidney disease population. Some items may not be relevant or may require different scoring approaches, which is particularly important since this represents the primary outcome.

Recommendations:

1. Reconsider the baseline data collection strategy. Either recruit during transplant evaluation or develop a robust plan for emergency consent and data collection that acknowledges the practical realities of the transplant setting.
2. Strengthen the statistical analysis plan. Collaboration with a biostatistician experienced in multi-omics integration would help develop a more sophisticated approach to multiple testing correction.
3. Clarify the group matching strategy and ensure it aligns with the stated goals of studying frail populations.
4. Consider adding an intermediate post-transplant time point to capture dynamic changes and address the missing data issue for specific biomarkers.
5. Provide more detail on the adaptation of the Frailty Index for the younger study population, including any modifications to scoring or item selection.

Recommendation: Minor revision - the concept is sound, and the work addresses an important question; however, methodological clarity and feasibility planning require improvement before publication.

VERSION 1 - AUTHOR RESPONSE

Reviewer 1 (Dr. Elizabeth Lorenz):

- Abstract

Group A is called group BA. Please correct

Response: Corrected throughout the abstract (page 3).

Clarify that participants in Groups A and B will be ≥ age 40

Response: Added to abstract Methods section "Group A (kidney transplant recipients aged ≥ 40 years; n = 50) and Group B (patients aged ≥ 40 years active on the kidney transplant waitlist; n = 50…" (page 3).

This nonrandomised study will determine associations, not causality or pathophysiology. Recommend rephrasing sections stating that causality or pathophysiology will be determined.

We have revised language throughout to reflect that we will examine associations and explore potential mechanisms. Key changes include:

- Abstract (page 3): Changed hypothesis wording to "it is hypothesised that frailty secondary to kidney failure and age-related frailty may be associated with similar molecular and physiological measures. These specific measures…"
- Introduction (page 10): "To gain a better understanding of frailty aetiology, this study will explore associations between"
- Throughout: Replaced "determine," "establish" with "explore," "examine," "assess associations"
- Discussion: "may uncover," "have the potential to identify"

Add brief description of planned statistical analyses

Response: Added to abstract Methods section (page 4): "Longitudinal changes in frailty will be analysed using linear mixed models. Associations between baseline frailty and molecular and physiological measures will be explored using multivariate regression with multiple testing corrections applied to control for false discovery rates."

Given that sample size was calculated using Frailty Index, consider identifying Frailty Index as primary outcome.

Response: We have now clearly designated the Frailty Index as the primary outcome in the new "Study Outcomes" subsection (page 9). The abstract and sample size calculation sections now explicitly identify FI as the primary outcome (Pages 3, 12).

- Introduction Issues

What is the difference between the third and fourth research questions?

Response: We have clarified the distinction (page 7):

- RQ3: "How do the molecular and physiological measure co-vary with the change in frailty status over the 12-month study period?"
- RQ4: "Are baseline (Time 0) molecular and physiological measures associated with frailty at 12 month follow up?"

RQ3 examines the co-variation of change. The independent variables are the change scores (changes in measures) in molecular and physiological variables over 12 months, correlated with the change score in frailty status (change in frailty) over the same 12 months.

RQ4 examines prospective association. The independent variables are the baseline values of the molecular physiological measures associated with the subsequent trajectory (or rate of change) in frailty status over the 12-month period.

This distinction allows us to explore two separate, critical relationships: how much change happens together (RQ3) versus what baseline factors influence future change (RQ4).

The authors state that frailty will improve Group A and worsen in Groups B/C. This is not necessarily true.

Response: Revised language to acknowledge heterogeneity (page 10): "Specific molecular and physiological measures are anticipated to improve in most transplant recipients (as frailty typically improves (2)), though we acknowledge that individual trajectories will vary, with some patients experiencing transient worsening or no change. Similarly, measures may worsen in patients who remain on dialysis or with age-related frailty, though responses will be heterogeneous." Figure 1 caption now states: "Trajectories represent anticipated mean changes; individual patients may experience improvement, no change, or worsening."

Outcomes are collectively referred to by different terms. Consider using the same phrase throughout.

Response: Standardised to "molecular and physiological measures" throughout when referring to secondary outcomes collectively (global change across manuscript).

Methods

- Study Design

Add age cut-offs for each group.

Response: Added to Study Design section (page 11): "Group A – adult patients with kidney failure who receive a kidney transplant (aged ≥40 years; n=50), Group B – adult patients of similar age to Group A (aged ≥40 years) with kidney failure who are active on the waiting list (n=50), and Group C – older adults without kidney failure (aged ≥65 years, or ≥55 years for Aboriginal and Torres Strait Islander patients; n=50)."

Why does it say Group C will be 'frailty-matched'? Please clarify.

Response: Removed "frailty-matched" terminology throughout and clarified (page 11): "Group C comprises older, community-dwelling adults who, based on previous studies (16, 21, 22) and clinical frailty scales of 2-6, are anticipated to have similar baseline FI scores to Groups A and B, enabling comparison of age-related with disease-related frailty."

- Eligibility

Why ≥ 40 years for A/B? Why ≥ 55 years for Aboriginal and Torres Strait Islanders in Group C?

Response Added justification in Eligibility section (pages 10 & 11); "Groups A and B – Kidney failure patients on the QKTS waitlist:

- Kidney failure (CKD stage 5 on dialysis, or pre-dialysis if pre-emptive living kidney donor recipient)
- Aged ≥ 40 years – chosen due to the increasing prevalence of frailty in kidney disease populations from this age (11, 12)."

And "Group C – older community-dwellers:

- Clinical Frailty Scale (CFS) of 2-6. Our recruitment goal is to capture a cohort of older people with a similar frailty status to Groups A and B. In our study of 147 patients with kidney failure attending the QKTAC, mean Frailty Index was 0.23 (SD 0.10). Australian community-dwelling older adults requiring geriatric assessment and support have a CFS between 2 and 6, with mean FI 0.23(13)
- Aged ≥ 65 years, or ≥ 55 years for Aboriginal and Torres Strait Island patients. For Group C, ≥65 years reflects the standard age threshold for geriatric assessment in Australia (14), while ≥ 55 years for Aboriginal and Torres Strait Islander patients recognises the well-documented earlier onset of age-related conditions in these populations, consistent with Australian clinical guidelines (15)."

Consider including 'anticipated to progress to transplant within 3 months' as eligibility criteria for Group A

Response: Added to inclusion criteria for Group A (page 10): "patients anticipated to progress to transplant within 3 months of enrolment based on blood group compatibility, position on waitlist, and clinical assessment."

Explain why frailty is not an eligibility criterion for Groups A and B while it is in Group C?

Response: Added clarification (page 14):

"Frailty is not a criterion for inclusion/exclusion for Groups A and B because frailty is highly prevalent in kidney transplant candidates (affecting 40-79% depending on the measure used; (16)), and based on previous data we anticipate the mean FI is 0.23 {Weerasekera, 2023 #1806}. Furthermore, we anticipate the FI of Group C to be similar."

Explain why Group A includes patients not on dialysis while Group B only includes patients on dialysis

Response: Added explanation (pages 10 & 11): "Group A includes both patients receiving pre-emptive, live donor transplants and those on dialysis receiving either live or deceased donor transplants, as both pathways are common in clinical practice and both populations experience frailty. In contrast, Group B is restricted to patients on dialysis, as renal replacement therapy is a requirement for waitlist eligibility."

- Sample Size

Was participant withdrawal planned for?

Response: Our sample size calculation targets 50 participants per group without inflation for participant withdrawal decision is based on statistical power requirements and anticipated retention rates specific to the clinical context.

Statistical power and paired design: Our primary research questions employ a paired design (within-group comparison of changes), which significantly mitigates the statistical impact of loss-to-follow-up compared to independent group comparisons (17). Our sample size calculation is powered to maintain adequate statistical power for the primary outcome with a minimum of 30 complete cases per group.

Justification for low anticipated attrition: We anticipate a high retention rate over the 12-month follow-up, supported by citable evidence from similar research cohorts:

- Kidney cohorts: participants are actively engaged in highly specialised transplant and nephrology services, involving regular clinical contact and high motivation for research related to their critical care pathway. This assumption is consistent with established, low-attrition rates in longitudinal studies conducted by the Australian Kidney Transplant Network (AKTN) (18).
- Older adults: Retention in our geriatric clinic attending cohort is anticipated to be high, reflecting the successful follow-up rates achieved in similar clinical settings (19).

Mitigation and analysis: All participant withdrawals and losses to follow-up will be reported transparently. Our primary analysis will utilise an intention-to-treat approach, with complete case analysis serving as a secondary sensitivity analysis to examine the effect of missing data. We acknowledge that a dropout rate exceeding our anticipated low rate represents a design limitation.

- Data Collection

Clarify whether samples will be taken prior to dialysis days in Group A.

Response: Clarified "For Group A deceased donor recipients on dialysis, blood samples will be drawn prior to transplant surgery during pre-operative procedures."

- Measures

Justify choice of Rockwood Frailty Index as primary outcome.

Response: Added new paragraph in Frailty Index subsection "The Frailty Index was selected as the primary outcome measure for several reasons. First, it has demonstrated responsiveness to change in longitudinal studies across several age-related, and chronic disease-related populations (51-53). Secondly, unlike phenotypic measures (e.g., Fried Frailty Phenotype) that focus primarily on physical frailty, the FI captures multiple health domains including cognitive function, nutrition, mood, and comorbidities providing comprehensive assessment relevant to the multisystem challenges expected with transplantation. Third, the FI has been validated in kidney failure patients attending transplant clinics in our previous work (24). Finally, the accumulation of deficits approach aligns with the conceptual framework that frailty represents multisystem decline, which we aim to characterise through molecular and physiological measures."

Provide Rationale for each study outcome

Response: We have expanded Table 1 to include brief rationales for each measure category. For example, under "Tissue Integrity" we added: "cf DNA and mtDNA are emerging biomarkers of cellular stress, tissue damage, and biological ageing, elevated levels of which have been associated with frailty and mortality."

- Data analysis

Describe how you will account for multiple hypothesis testing and baseline differences

Response: Substantially expanded data analysis section to include: "To address the challenge of multiple hypothesis testing across high-dimensional omics data, we will implement the following strategies: (1) False Discovery Rate (FDR) control using the Benjamini-Hochberg procedure will be applied within each omics platform; (2) principal component analysis will be used to identify independent data dimensions with the effective number of tests determined by the number of principal components explaining >99% of variance; (3) For exploratory analyses, we will report both uncorrected and FDR-corrected p-values with appropriate caveats; (4) Key findings will be prioritised for validation in future studies. Baseline differences among groups will be addressed by including relevant covariates (age, sex, comorbidities, medications and diet) in multivariate models and by reporting both adjusted and unadjusted analyses. We recognise that the sample size relative to the number of potential biomarkers limits our ability to conduct definitive hypothesis testing. This study is designed to be exploratory and hypothesis-generating, identifying promising biomarkers and pathways for focused investigation in larger, confirmatory studies."

Is FI only analysed as continuous variable?

Response: Added (page 16): "The FI will be analysed primarily as a continuous variable to maximise statistical power and capture subtle changes. In secondary analyses, we will stratify the FI using established thresholds (e.g., $FI \leq 0.10$ = non-frail, $0.10 \leq FI \leq 0.21$ = pre-frail; $FI > 0.21$ = frail) to facilitate clinical interpretation and comparison with existing literature."

- Table 1

Consider moving Table 1 from Study Design to Measures section. Include complete list of all measures.

Response: Moved to Table 1 to Measures section (page 17 & 18) and expanded to include complete lists rather than examples. For instance, "Health Indicators" now lists: "Circulating

inflammatory markers (IL-6. CRP), kidney function blood tests: serum creatinine, eGFR, urea, albumin"

- Figure 1

Consider changing to study flow figure rather than hypothesised trajectories.

Response: We have retained the trajectory visualisation as it illustrates our core conceptual hypothesis, but have modified the caption substantially: "The plotted trajectories represent anticipated mean changes based on findings in published literature (2, 20-22). While these slopes show the expected group averages, individual patient trajectories (i.e., improvement, no change, or worsening). Actual, empirically derived trajectories and their confidence intervals will be reported in the study results."

Reviewer 2 (Dr Andrea Carla Bauer)

- Pre-transplant Data Collection

The biggest concern is the plan to collect baseline data "just before transplantation" for deceased donor recipients. This presents significant logistical challenges that may compromise data quality.

Response: We acknowledge this is a legitimate concern. However, recruitment has already commenced using the described protocol, and we have successfully collected baseline data from multiple participants in this situation. While challenging, this approach is feasible and scientifically necessary for several reasons:

1. Timing feasibility: At our centre, there is typically a window of several hours between patient arrival and surgery during organ preparation and preoperative procedures. Research staff coordinate with both ward staff and the surgical team to collect data during this window.
2. Streamlined procedures: Blood draws occur during routine pre-operative blood work. The FI interview takes 10-15 minutes. Microcirculation assessment takes ~15 minutes and is non-invasive. Data required to be collected pre-surgery (e.g., blood

draws, microcirculation assessment) are prioritised and interviews/surveys are completed retrospectively if time does not permit.

3. Flexibility for stool samples: Our protocol states "if possible" for pre-transplant stool samples, acknowledging this may not always be feasible for deceased donor recipients. We have provided contingency for stool sample collection up to 48 hours post-surgery, where time of collection is acknowledged.
4. Live donor recipients: Approximately 30-40% of Group A will be live donor recipients with scheduled surgeries, allowing controlled baseline collection.
5. Scientific rationale: Collecting data during initial transplant evaluation (as suggested) would create significant problems: many evaluated patients never receive transplants, time between evaluation and transplant can be years, and frailty status changes substantially during waiting, making it impossible to establish a true baseline before surgery.

We have added to Strengths and Limitations (page 5): "Baseline data collection for deceased donor recipients occurs during the pre-operative period, which may be stressful and may result in some missing data for certain measures (e.g., stool samples); however, pilot experience demonstrates this is feasible for most core measures."

We have also added to Data Collection section (page 13): "We acknowledge the logistical challenges of baseline data collection immediately before deceased donor transplantation. However, this timing is essential for capturing true baseline frailty status pre-transplantation. Alternative approaches (such as collecting baseline during transplant evaluation and within 24 hrs after surgery) introduces substantial time for data collection without confounding before-after comparisons."

Our reasoning for this methodology is that consultation with consumers with lived experience of kidney transplantation during study design deemed the collection strategy appropriate.

Consumers agreed with the acknowledgement of logistical challenges, however did not believe it warranted methodological changes.

- Statistical Analysis

The proposed solution for multiple testing appears inadequate. With 50 patients per group and potentially thousands of biomarkers, a serious multiple testing problem will arise.

Response: We have substantially expanded our statistical analysis section (see response to reviewer 1 above, pages 27-28). Key additions include:

- FDR control within each omics platform
- PCA-based dimensionality reduction
- Clear distinction between hypothesis-testing and exploratory analyses
- Explicit acknowledgement that the study is primarily hypothesis-generating

We have also added (page 26): "We recognise that the sample size relative to the number of potential biomarkers limits out ability to conduct definitive hypothesis testing. This study is designed to be exploratory and hypothesis-generating, identifying promising biomarkers and pathways for focused investigation in larger, confirmatory studies."

Furthermore, the longitudinal repeat measures design will improve power as it will allow us to perform within-individual comparison instead of performing group comparisons which are less powerful as they do not account for baseline inter-individual differences.

Sample size calculation assumes improvement from 0.23 to 0.06, which may be overly optimistic.

Response: We acknowledge this concern. The expected improvement to FI = 0.06 is based on published data showing mean FI in the general population for this age group is 0.06, and studies showing physical frailty in transplant recipients approaches age-matched healthy control levels by 12 months (2, 9). We have added (page 16): "This represents a scenario assuming substantial frailty improvement. If actual improvement is more modest (e.g., from 0.23 to 0.15), power for between-group comparisons would be reduced. However, our primary analysis focuses on within-group changes using paired analyses, which have greater statistical power than between-group comparisons. Additionally, we would retain >80% power to detect a more conservative between-group difference of 0.08 FI units"

- Group Matching Strategy

The frailty-matched terminology is unclear and matching strategy is not explained

Response: As addressed above (Reviewer 1, Editor comments), we have removed 'frailty-matched' terminology and clarified that we anticipate similar baseline FI scores based on epidemiological data and data from our group, without formal matching.

Group C uses CFS 2-6, which includes essentially well people (CFS 2).

Response: Please see response to Reviewer 2.

Our recruitment goal is to capture a cohort of older people with a similar frailty status to Groups A and B. In our study of 147 patients with kidney failure attending the QKTAC, mean Frailty Index was 0.23 (SD 0.10). Australian community-dwelling older adults requiring geriatric assessment and support have a CFS between 2 and 6, with mean FI 0.23(13),

The age difference (≥40 vs ≥65) complicates matching and requires justification

Response: We have added justification (page 14, see above) and added to discussion (page 31): "The inherent age difference between the patient groups and the control cohort represents a necessary design constraint to test the hypothesis of shared frailty mechanisms across the lifespan. However, this is also a methodological limitation that must be carefully addressed. We will include age as a covariate in all between-group and longitudinal statistical models to account for its independent effects on molecular and physiological measures. This statistical mitigation is critical for ensuring that the final data, our expected outcomes, allow us to isolate the specific effects of the disease state from the normal aging process. The robustness of this statistical approach is vital for the eventual clinical relevance of our findings."

- Missing Post-Transplant Measurements

Inability to collect microcirculation and microbiome immediately post-transplant creates data asymmetry

Response: We agree this creates asymmetry, but early post-transplant collection would not be scientifically valid:

- Microcirculation – acute haemodynamic changes, anaesthesia effects, and fluid shifts would confound interpretation

- Microbiome: post-transplant antibiotics dramatically alter microbiome, making it impossible to separate transplant effects from medication effects. Please note that we have altered the protocol to allow for collection of stool samples up to 48 hours after transplantation post-transplant as a contingency to address significant asymmetry.

We acknowledge that an additional 6-month time point would be scientifically interesting, but this is not feasible given budget constraints, participant burden, and that recruitment has commenced.

- Frailty Index Application

The FI was developed in older adults. More discussion needed on adaptation for younger kidney disease population.

Response: We have added discussion (see Measures justification above). Additionally, we note that our research group has previously validated the FI-SF specifically in kidney failure patients across age ranges <65 years (23). We have added: "The Frailty Index was selected as the primary outcome measure for several regions. First, it has demonstrated responsiveness to change in longitudinal studies across several age-related, and chronic disease-related populations (24-26)."

- Summary

We have addressed all reviewer and editor comments. Where suggested methodological changes were not feasible due to commencement of recruitment, we have provided clear scientific rationale and acknowledged limitations appropriately. We feel that these clarifications and additions have strengthened the manuscript.

We hope you will find our revisions acceptable and look forward to hearing from you.

1. McAdams-DeMarco MA, Law A, Salter ML, Boyarsky B, Gimenez L, Jaar BG, et al. Frailty as a novel predictor of mortality and hospitalization in individuals of all ages undergoing hemodialysis. J Am Geriatr Soc. 2013;61(6):896-901.
2. McAdams-DeMarco MA, Isaacs K, Darko L, Salter ML, Gupta N, King EA, et al. Changes in Frailty After Kidney Transplantation. J Am Geriatr Soc. 2015;63(10):2152-7.
3. Chu NM, Chen X, Norman SP, Fitzpatrick J, Sozio SM, Jaar BG, et al. Frailty Prevalence in Younger End-Stage Kidney Disease Patients Undergoing Dialysis and Transplantation. Am J Nephrol. 2020;51(7):501-10.

4. Perez-Zepeda MU, Godin J, Armstrong JJ, Andrew MK, Mitnitski A, Kirkland S, et al. Frailty among middle-aged and older Canadians: population norms for the frailty index using the Canadian Longitudinal Study on Aging. Age Ageing. 2021;50(2):447-56.
5. Verschoor CP, Theou O, Ma J, Montgomery P, Mossey S, Nangia P, et al. Age- and sex-specific associations of frailty with mortality and healthcare utilization in community-dwelling adults from Ontario, Canada. BMC Geriatrics. 2024;24(1):223.
6. Elhussein L, Robinson DE, Delmestri A, Clegg A, Prieto-Alhambra D, Silman A, et al. Longitudinal trajectories of frailty are associated with short-term mortality in older people: a joint latent class models analysis using 2 UK primary care databases. Journal of Clinical Epidemiology. 2024;173:111442.
7. Donnoli C, De Luca V, Formosa V, Picardo G, Aprile A, Carnevale E, et al. Frailty and quality of life in italian older adults: implications for public health interventions. European Journal of Public Health. 2024;34(Supplement_3).
8. Weerasekera S, Reid N, Young A, Homes R, Sia A, Giddens F, et al. Putting Guidelines Into Practice: Is Frailty Measurement at the Time of Kidney Transplant Eligibility Assessment Valid, Feasible, and Acceptable to Patients? Transplantation Direct. 2023;9(11):e1548.
9. Loecker C, Schmaderer M, Zimmerman L. Frailty in Young and Middle-Aged Adults: An Integrative Review. J Frailty Aging. 2021;10(4):327-33.
10. Theou O, van der Valk AM, Godin J, Andrew MK, McElhaney JE, McNeil SA, et al. Exploring Clinically Meaningful Changes for the Frailty Index in a Longitudinal Cohort of Hospitalized Older Patients. J Gerontol A Biol Sci Med Sci. 2020;75(10):1928-34.
11. Chowdhury R, Peel NM, Krosch M, Hubbard RE. Frailty and chronic kidney disease: A systematic review. Arch Gerontol Geriatr. 2017;68:135-42.
12. Chu NM, Chen X, Norman SP, Fitzpatrick J, Sozio SM, Jaar BG, et al. Frailty Prevalence in Younger End-Stage Kidney Disease Patients Undergoing Dialysis and Transplantation. American Journal of Nephrology. 2020;51(7):501-10.
13. Khadka J, Visvanathan R, Theou O, Moldovan M, Amare AT, Lang C, et al. Development and validation of a frailty index based on Australian Aged Care Assessment Program data. Med J Aust. 2020;213(7):321-6.
14. Garrard JW, Cox NJ, Dodds RM, Roberts HC, Sayer AA. Comprehensive geriatric assessment in primary care: a systematic review. Aging Clinical and Experimental Research. 2020;32(2):197-205.
15. Cotter PR, Condon JR, Barnes T, Anderson IPS, Smith LR, Cunningham T. Do Indigenous Australians age prematurely? The implications of life expectancy and health conditions of older Indigenous people for health and aged care policy. Australian Health Review. 2012;36(1):68-74.
16. Kao J, Reid N, Hubbard RE, Homes R, Hanjani LS, Pearson E, et al. Frailty and solid-organ transplant candidates: a scoping review. BMC Geriatr. 2022;22(1):864.
17. Austin PC. Comparing paired vs non-paired statistical methods of analyses when making inferences about absolute risk reductions in propensity-score matched samples. Stat Med. 2011;30(11):1292-301.
18. King SJ, Reid N, Brown SJ, Brodie LJ, Sia ADH, Chatfield MD, et al. A prospective, observational study of frailty, quality of life and dialysis in older people with advanced chronic kidney disease. BMC Geriatrics. 2023;23(1):664.
19. Kawai H, Ejiri M, Tsuruta H, Masui Y, Watanabe Y, Hirano H, et al. Factors associated with follow-up difficulty in longitudinal studies involving community-dwelling older adults. PloS one. 2020;15(8):e0237166.
20. McAdams-DeMarco MA, Olorundare IO, Ying H, Warsame F, Haugen CE, Hall R, et al. Frailty and Postkidney Transplant Health-Related Quality of Life. Transplantation. 2018;102(2):291-9.
21. Chu NM, Gross AL, Shaffer AA, Haugen CE, Norman SP, Xue QL, et al. Frailty and Changes in Cognitive Function after Kidney Transplantation. J Am Soc Nephrol. 2019;30(2):336-45.
22. Homes RAP, Giddens F, Francis RS, Hubbard RE, Gordon EH, Midwinter MJ. The sublingual microcirculation and frailty index in chronic kidney disease patients. Microcirculation. 2023:e12819.

23. Hubbard RE, Peel NM, Smith M, Dawson B, Lambat Z, Bak M, et al. Feasibility and construct validity of a Frailty index for patients with chronic kidney disease. Australas J Ageing. 2015;34(3):E9-12.
24. Lai JC, Sonnenday CJ, Tapper EB, Duarte-Rojo A, Dunn MA, Bernal W, et al. Frailty in liver transplantation: An expert opinion statement from the American Society of Transplantation Liver and Intestinal Community of Practice. Am J Transplant. 2019;19(7):1896-906.
25. Varughese RA, Theou O, Li Y, Huang X, Chowdhury N, Famure O, et al. Cumulative Deficits Frailty Index Predicts Outcomes for Solid Organ Transplant Candidates. Transplant Direct. 2021;7(3):e677.
26. Fletcher J, Reid N, Hubbard RE, Berry R, Weston M, Walpole E, et al. Frailty Index, Not Age, Predicts Treatment Outcomes and Adverse Events for Older Adults with Cancer. J Frailty Aging. 2024;13(4):487-94.

VERSION 2 - REVIEW

**Reviewer**	**1**
**Name**	**Lorenz, Elizabeth**
**Affiliation**	**Baylor College of Medicine**
**Date**	**18-Nov-2025**
**COI**	

The authors addressed my comments.
